# Out There No One Has a Right to Die

**DOI:** 10.1111/bioe.70083

**Published:** 2026-01-21

**Authors:** Matti Häyry

**Affiliations:** ^1^ Aalto University School of Business Espoo Finland

**Keywords:** colonialism, ethics, intergenerational, space exploration, totalitarianism

## Abstract

The eventual goal of space exploration is to colonize exoplanets and their moons outside our solar system. This is a dangerous and immoral endeavour. The extraterrestrial life forms encountered would be hostile, vulnerable or both, and the descendants of the original pioneers would be involuntarily exposed to hazardous conditions and totalitarian regimes. They would have little or no control over their lives, reproduction, or death. Even in the unlikely event that *homo sapiens*, the species responsible for degrading its own home planet, could be expected to fare better in a new habitat in the future, the sacrifices thrust upon the non‐consenting interim generations would be ethically unacceptable.

## 1 About the Approach

Human activities sometimes involve a threat of serious harm to human wellbeing, although the likelihood of the risk is scientifically uncertain. In other words, potential harm is identified but conclusive scientific evidence is lacking, making it impossible to fully evaluate the quantity of the risk. In such situations, the precautionary principle advises us to engage in these activities only when it has been established that the risk is sufficiently low to be managed by feasibly executable control arrangements and mechanisms. According to the principle, the burden of proof lies with those who wish to initiate or continue the activities. The presumption against them must be reasonably comprehensible—but it does not require detailed justification.

This paper applies the precautionary principle to space exploration and travel. It presents a landscape of dangers which is reasonably comprehensible to relatively cautious, liberal individuals who do not trust technological developments without question. It plants the burden of proof in the corner of those who want to explore, exploit and populate outer space. If the dangers can be overcome, let that be shown, scientifically and conclusively. This paper alludes that the task may be too difficult to be completed.

This non‐exhaustive and mainly speculative exposition proceeds from a specific position. Views concerning justice, or political morality, can be roughly divided into four main categories. Following my own prior classification and using a relatively conventional nomenclature, I call these conservative, liberal, progressive, and radical [1, p. 206 Figure 11].^1^ My treatise here concentrates on a ‘radical’—traditionally utilitarian—criticism of what I see as ill‐conceived ‘liberal’—in this case, enthusiastically techno‐reliant—preconceptions concerning the alleged advantages of future space travel. Representatives of these two views, with their similarities and their differences, are the ‘we’ of my narrative.

In this juxtaposition, both parties share many anthropological, social, and political assumptions: individualism, limited altruism, freedom as nonrestriction, and so on. They can also operate at a fairly abstract and fictional level, drawing their technological and futurological imagery from science fiction, including comic books. Their difference, though not sharply defined, is nonetheless marked: from the radical critic's perspective, it lies in the liberals' unwarranted trust in the Enlightenment ideas of human malleability and social progress.

In light of this starting point, I endeavour, in what follows, to present an informal radical criticism of what I see as unwarranted liberal hopefulness concerning the future of space travel. This will culminate in the case signalled in the title—the right to die—about which both the liberals and the radicals in my framework are uncertain but cautiously supportive. They may not advocate an absolute, state‐enforced licence to have one's life ended at will, but neither do they reject the idea out of hand. This uncertainty is all I need for my account.

The deliberate limitation of my approach has two major implications that deserve to be noted. The first is that the more non‐fictional—technical or scientific—takes on future space travel and its implications are bracketed. I have compensated for this by including references to recent works on these perspectives as the story unfolds. The second implication of the limitation is that the ‘conservative’ and ‘progressive’ views on future space travel and its ethics are also bracketed. References to current literature have been added to keep them, albeit indirectly, in the frame.

The upshot of these methodological choices is that the primary audience for my ruminations comprises liberals and radicals who have a stake in the ongoing debate on space ethics. For those who do not share the—essentially individualistic—assumptions, or the imagery of the turn‐of‐the‐millennium science fiction, my considerations can only offer insights into the modes of thought shaping this ideological terrain.

## 2 Setting the Scene

Envisioning space travel may, to the specified ‘we’ of my account, feel as mundane as imagining a ride in a self‐driving car from the city centre to one of its surrounding neighbourhoods. This comparison holds especially true when considering the external factors that prompt such a decision and define its outer limits. The internal factors—the ones operative during the ride—are another matter entirely. Yet on both dimensions, alarm bells should be ringing.

Let us begin with a snapshot of the external dimensions. Why leave the centre? Perhaps because it no longer meets a critical need. Which neighbourhood should we choose? Reasonably, the one that promises to provide what we lack. But what if we don't know which neighbourhood that is? Depending on the urgency, we might stay put or make a random choice and hope for the best. Would the well‐being of the inhabitants at our destination factor into the decision? Perhaps, if we're exceptionally ethical—but more likely, no. That's a concern for later. And if they're hostile, ready to defend their territory? No worries—we'll pack some armament, just in case. All good and familiar. Explorers have done that for ages. We are set to take our trip.

What about the internal factors, then? We can imagine taking a trip in a self‐driving car even if we've never experienced it.^2^ After all, we've ridden in vehicles not driven by ourselves. The interior would likely be similar to other cars we've encountered. But outer space voyages? That's another matter entirely. Apart from the technology—another huge consideration—the mention of explorers offers a clue often overlooked in discussions of interstellar travel. Here's a simple test question: what were the conditions like on board the Niña, Pinta and Santa Maria? Did they resemble the comfort of your car? Of course not. The gap, popular science‐fiction imagery notwithstanding, could be immense, and this must be factored into any serious contemplation of space travel.

In what follows, I will explicate how these observations could—and perhaps should—shape the bioethics of space exploration. Are we justified in travelling to alien neighbourhoods to meet our own needs at the expense of others? This is a fundamental question of justice, applicable on Earth as in heaven. And what would our lives—physical, social, and political—be like on board a spacecraft, possibly as a part of a unified fleet? To what extent would our health, autonomy, and rights matter to the community? Whose benefit would be the measure of all things?

## 3 Reasons for Space Exploration

Why would the human race, or any other species anywhere, want to travel to other planets or to other solar systems? I will consider more mundane—and potentially more sinister—options in due course, but literary science fiction and superhero comics have taught that one of the better reasons is a home planet becoming uninhabitable. Superman is sent to Earth by his parents because Krypton is dying. The survival of the species necessitates departure—or, in his case, the nonconsensual deportation [2]. These are the half‐conscious mental images shaping the views of my liberals and radicals.

Assuming there are costs to the travellers, to indigenous species elsewhere, or to both, as in the Superman saga, a further question becomes pertinent: is leaving the original habitat forced upon the space invaders by external factors beyond their control, or might there be alternative ways of solving the possibly self‐induced problem? The situation on Krypton varies in different stories, but it is plausible that it is exploding simply due to old age [3]. That, in the mindset of my stipulated ‘us’, could justify rescue operations, even at some cost.

Using the same justification for human space exploration is not straightforward. Our planet may well be dying [4], and something should presumably be done about this [5], but abandoning Earth, tempting as it may seem [6, 7], is neither a very responsible nor an assuredly just and sustainable solution [8]. Why does humankind not solve its self‐inflicted problems by itself, with the resources in its command now? This would, of course, require sacrifices—less people, less consumption [9], and so on—but as long as domestic solutions remain a possibility, exploiting other parts of the universe does not seem like the fairest response. Colonialism has a nasty ring to it.

Evoking the Lockean proviso does not help here [10, p. 132],^3^ although the idea lurks behind all visions of space invasion [11]. According to it, we either enter uninhabited territories unusable to anyone else or leave others as much and as good, thereby causing no issues of distributive justice. Global capitalism has shown the hollowness of the as‐much‐and‐as‐good pipe dream all around the world. As for the uninhabited‐and‐unusable‐to‐others disclaimer, a look at where we might be going reveals that it is probably not tenable.

## 4 Spaces to Explore

The initial plans made by the United States (U.S.) government and supported by contemporary billionaires are confined to our own solar system, Mars being the preferred target for the most imminent colonialization [12, 13]. The Moon, due to its relative proximity, provides in the plans a training ground for adjusting the travel arrangements and living conditions off Earth [14].

Some of the stated goals suggest that, at least initially, Mars and the Moon would be exploration and mining sites rather than permanent homes for the travellers. Even in the longer term, they are unlikely destinations for pioneers looking for sustainable living conditions over generations. They expose their possible inhabitants to hostile environments in constant need of observation and control, resembling hazardous working conditions in extreme heights, depths, heat or cold more than just challenging unfamiliar farming grounds on the frontier lands of the past.

With the relatively limited resources of the Moon and Mars, the sustainability of immigrant life could also become an issue. Humankind, after its population expansion over the last centuries, has quickly depleted one planet's natural riches and may do the same with other supposed havens. This is why the eventual strategy on the horizon must be to scale up and reach beyond our solar system, to territories with virtually endless opportunities.

At this point, the question of ‘when’ seems to fade into the background. The U.S. National Aeronautics and Space Administration (NASA) has deployed robots of varying capabilities to Mars since 1976. However, even the most enthusiastic billionaires estimate that a human mission is still decades away. And Mars, while a significant milestone, represents only a small step towards the advanced interstellar visions of science fiction. Achieving such ambitions will require efforts spanning multiple generations.

Moving on, for argument's sake, to the possibility of exiting our solar system and entering others, what would we be looking for? Obviously, areas which would be liveable to homo sapiens and other Terran DNA without cumbersome protective gear. The entertainment business, notably exemplified by the *Star Trek* television and movie saga, has given us the imagery of foreign planets on which we can breathe and move as naturally as on Earth. Either the conditions spontaneously suit us, or we can adjust to new conditions with proper equipment or enhancements [15].

## 5 Encountering the Vulnerable

While it is unrealistic to believe that the first habitable planet we encounter outside our solar system is an Earth double, it is, by definition, habitable. But if we can live there, so can others, and we may not be the first ones to discover the territory. What, then, of those who came before us?

One possibility is that their strength and skills are so inferior to ours that we could easily subjugate them. This scenario is disturbingly familiar: it echoes our instrumental treatment of nonhuman animals and the actions of conquistadors and their descendants toward indigenous peoples worldwide.

A choice must be made. Will we act as egoistic speciesists, indifferent to the plight of the original inhabitants? Or, as one hopes, can we find a morally more palatable approach? Armed with bioethical principles and values [16], we can outline an answer to the latter question. It would be wrong to harm them, right to help them, imperative to respect their self‐determination, and essential to treat them justly [17]. We should also honour their autonomy, integrity, and vulnerability while demonstrating solidarity [18]. Admirable ideals—and standardly held by the liberal and radical ‘us’ of my narrative—but what would our probable course of action in reality be?

Absent significant moral progress, ‘our’ approach is likely to prioritize self‐interest under the guise of paternalistic benevolence. If the indigenous inhabitants are sufficiently advanced to comprehend it, this strategy might also include ideological conditioning framed as guidance or improvement. Justice would be framed in utilitarian terms and autonomy conceived as submission to our rational rule. Any voices of fundamental otherness would be ignored and, if need be, demonized. In other words, business as usual in the ever‐expanding sphere of global capitalism [19]. This is what ‘we’ have always done.

A more genuinely ethical approach is, of course, conceivable [20]. One option—and this is my personal ‘radical/liberal’ outtake of my exposure to applied moral and political philosophy—is a hybrid consequentialism that abandons simple utility aggregation and allows a more nuanced touch in conflict situations [21]. It could be based on a realization that ‘we are one with all other sentient beings, and that we should not by our actions or choices make their lot worse;’ [22] and that ‘other living beings have their own life directions and [we] should not impose [our] own ways of thinking and being upon them.’ [23] These realizations—which as attitudes I have called ‘copathy’ and ‘dissense’—could be conducive to a kinder take on relationships with alien life forms. We share our vulnerability with them, and they have their own goals and destinies. Let us be kind. Let us not be unkind. That, in all its simplicity, could do the trick. It is just that humankind is unlikely to follow even these simple instructions if its own more immediate preference satisfaction is at stake.

## 6 Encountering the Hostile

During our space explorations, whether we find habitable environments or not, we may come across extraterrestrial intelligence that is superior or equal to ours. If that happens, we need rules for the first contact lest we blow our chances of collaboration or even survival.^4^

It is a question of its own, of course, whether extraterrestrial consciousness exists and what it might be like. Enrico Fermi famously pondered the discrepancy between the apparent probability of intelligent life in the universe and the lack of evidence for it. ‘Where is everybody?’ Fermi's Paradox asks [24]. One optimistically antinatalist answer is: ‘Already wisened up and gone.’ This response reflects the hope that civilizations recognize their cosmic futility before they develop technologies capable of spreading and wreaking havoc among distant cultures [20, p. 31]. Another conjecture, by contrast, is the dark forest hypothesis, which holds that alien civilizations, hostile but silent, may abound throughout the universe but remain undetectable out of fear of annihilation by others [25].

The notion of a Hobbesian trap in the literature on first encounters has it that since sufficient, trust‐inspiring communication with alien intelligence is impossible, we should be prepared to resort to the use of pre‐emptive military force [26]. This bleak picture of interstellar relations is based on the idea that both we and the intelligent extra‐terrestrials make our decisions game‐theoretically, calculating the gains and threats as anti‐coordination games like Chicken and Hawk‐Dove [27]. It has been argued, against this, that a mutual understanding of intentions can, despite initial challenges, be possible, and that taking a hostile attitude might not be the wisest strategy with potentially dangerous strangers [28].

The message is further amplified by considerations of the nature of possible forms of reactive and conscious beings in other galaxies and solar systems. In our anthropocentrism and anthropomorphism, we are prone to think that extraterrestrial life forms would be essentially like us—feed, bleed, think, believe, and feel pleasure and pain.^5^ Take away any one of these, and we are in truly uncharted waters. Superior beings without sentience, for instance, could see us as interesting but morally insignificant insects [29]. Approaching them hat in hand could be the second‐best option. Staying at home would be the best.

Then, again, we might encounter physically similar beings of approximately equal strength, which was the case with the European colonization of other continents. That the subsequent wars in our near history were won by the invaders was, to a considerable extent, due to diseases brought to the conquered territories by the intruders [30, 31]. Unscrupulous explorers might think of this as an asset. Ethically, of course, the mere thought is preposterous. We should not travel to outer space with the intent of spreading illnesses [32]. ^6^

Given the choice of new habitats, humankind would spontaneously opt for an atmosphere and environment liveable for their own ilk. This makes the consideration of exported diseases relevant—if life forms already exist in the chosen location, they may well be vulnerable to our ailments, as we may be to theirs. Even without explicit warfare, a battle of physiques could be in the cards. But maybe our medicine is so far advanced that we can cure anything we encounter. And perhaps, despite initial hostility, we can find a way to live in peace with other species in the outer space. The *Star Trek* saga amply illustrates both hostilities and amenities between vastly different kinds of entities. If only real life were like that.

## 7 First‐Generation Consent

Thinking about space exploration from the viewpoint of purely human bioethics,^7^ the first port of call is consent. In any liberal democratic setting, autonomy, liberty, freedom and personal self‐determination (some of these overlap but none are equivalent) [33] form the backbone of the relationship between the individual and the collective. Consent, having the individual's considered permission to procedures, is the most convenient and authority‐transferring way to operationalize what the principles stand for.

The fundamental assumption,^8^ then, is that all those participating in space exploration are doing so voluntarily and have explicitly expressed their acceptance. This is not a problem in current aeronautics administration—competent candidates are queuing to be recruited for extra‐terrestrial missions. The bouts in space have also, so far, been relatively short, and changes of heart during the mission have not been reported. Even the Boeing Starliner astronauts who in July 2024 became stuck in their orbital station for an undefined period seemed to be fine with their situation [34].

The Moon, with our current technology, is so close to the Earth that it takes only a few days to travel there [35]. Moon colonizers with cold feet could, at least in theory, return to Earth as quickly as—or quicker than— Arctic or Antarctic scientists from their observation points. Mars is farther away, with an estimated one‐way travel time of six to nine months [36]. That makes the commitment stricter but still palatable: with a limited span at the destination, a total duration of a couple of years is not extraordinary for work contracts in unusual and challenging circumstances. Given, however, that Mars and the Moon are only baby steps in long‐term plans for space conquest, the subsequent stages present a more serious problem for the liberal ethos.

John Stuart Mill famously argued that although individuals should otherwise be free to do what they genuinely want, voluntary slavery is an exception [37, pp. 101–102].^9^ We are not allowed to interfere with each other's voluntary actions, because we must respect each other's liberty. By selling themselves—or allowing others to sell them—as slaves, individuals would abdicate that liberty and undermine the very purpose of respecting their choices. This is why they can legitimately be prevented from doing so, despite the apparent restriction.

Long‐term space travel could easily be compared to selling oneself to slavery. Once the decision to join an intergenerational exploration party has been made and the vessel launched, there is no returning to Earth, and no regaining the freedom of movement and action the travellers once enjoyed on their home planet. They have irreversibly given up their liberty—and a liberal society could justifiably hold them back, preventing them from alienating that liberty in exchange for an unknown future. Moreover, they are doing this not only for themselves but also for their prospective progeny. Even if individuals were allowed to sell themselves into slavery, what about their descendants?

## 8 Second‐Generation Totalitarianism

When parents voluntarily emigrate to other countries or continents, their children are often obliged to come along to keep the family together. The arrangement, however, is reversible: the parents can decide to move back,^10^ or the children, once mature enough to make their own decisions, can choose to return to their country of departure. The original choice commits no one to a lifetime in an alien environment.

With intergenerational space exploration, the situation is different. Colonizers can return from the Moon or from other planets in our solar system. But once the fleet has set sail for other stars, the distance alone becomes an insurmountable barrier. Travel far enough, and even if a return were permitted, it could not be completed within a lifetime. Here, popular science‐fiction imagery can be misleading. In *Star Trek* and other ‘final frontier’ stories, humankind always develops faster‐than‐light travel that keeps Earth within shouting distance. When the United Federation of Planets or its Starfleet summons ships from deep space, they can usually make the trip to Paris or San Francisco in a matter of days [38]. In reality, based on our current knowledge, this will not be the case.

Another illusion fostered by comforting television series is the prevalence of democratic political systems in outer space. The creators of *Star Trek* have, interestingly, offered an alternative to this—the authoritarian Terran Empire of the Mirror Universe [39]. The main narrative, however, assumes that decisions are taken collectively in a liberal and participatory spirit, give or take the captain's technical authority in battlefield situations. This may be grossly unrealistic, given how communities under sustained stress—such as the pressures of a hostile space environment—tend to be organised. In such conditions, strict discipline and a measure of authoritarianism are likely to creep into the arrangement.^11^

Whatever the system turns out to be, the case can be made that members of the first generation are not unduly forced or coerced, given convenient definitions of force and coercion [40]. Maybe people should be allowed to sell themselves into slavery, or to conditions resembling it. But for intergenerational missions, they would also have to commit their descendants to the same fate. In a sense, of course, parents are doing this to their children on a regular basis, bringing them into life and then persuading them to perpetuate the reproductive cycle. This practice can be criticized but the criticism has not received wide popularity [41]. In the case of space exploration, however, the situation is open for reassessment. It may be one thing to commit your offspring to a world we know and, to an extent, control; yet another to condemn them to the eternal darkness of the outer space, to put the matter poetically.

## 9 Reproductive Heteronomy

Those going out on longer missions might seek to avoid allegations of atrocity by arguing that either further generations will not be needed, or that life for any who are born will be so blissful that they could not rationally withhold retrospective consent. I think they would be wrong.

Methods of bypassing procreation could include immortality [42], or more precisely considerable longevity [43], cryopreservation, and a total or partial emigration to virtual reality [44]. If successful, any one of these could remove the need to multiply during space missions. People living a 1000 years would have the time to travel relatively far and back, especially if their vital functions were halted for the interstellar legs of the journey, and computerized existence could make the arrangement even more sustainable.

Since all these are at their experimental stages, however, with no clear prospect of success, plans based on them are mostly just wishful thinking. It is quite rational, at least according to standard utilitarian thinking [42, pp. 28–31], to envision futures where as‐of‐yet non‐existing technologies can be reasonably expected to be in place—but irrational fears and hopes are a different matter altogether, and not reasonably applicable to decisions under uncertainty, as demonstrated in discussions on the shortcomings of science‐enthusiastic forms of consequentialist thinking [42, pp. 190–193] [45].

The success of an interstellar mission requires, then, reproduction, lest the spaceship be a lifeless morgue when it arrives at its destination. And taking into account the special circumstances, this reproduction must be strictly regulated, perhaps on the lines sketched by Plato's *Republic* [46]. Only the best offspring would be produced, quality being defined by the ability to perform designated functions on board. Potential producers of inferior offspring would be discouraged, and their progeny would be discarded before or shortly after their birth. Matches between potential producers of good offspring would be encouraged. The principle of reproductive heteronomy would replace the liberal democratic ideal of procreative autonomy.

In case of declining birth rates and reluctance to have children, women could be ordered to have them [47]. It is easy to imagine the plot of the anarcho‐feminist movie envisioning the situation. The protagonist, forced to pregnancy and labour, knowing how her mother near‐fatally fractured her space‐brittle pelvis in giving birth to her. Ectogenesis [48], a theoretical possibility in both cases, was ruled out because the only artificial wombs are in the mothership (!) and serve only members of the elite.^12^

So, not only would subsequent generations of space travellers be committed to life in the darkness but also to an illiberal political order with no respect for their privacy and self‐determination. Even as a possibility, this should warn off the liberals and radicals of my story.

## 10 Life and Death in Transition

The prospective disrespect is not confined to matters of propagation. Any control people have over the perpetuation and termination of their existence on Earth would effectively be cancelled out there. An individual's will to live or to die would be subordinated to the common good of the fleet and its mission.

The picture painted by science fiction, or its surface interpretation, is, once again, deceptive here. In the *Star Trek* movie *The Wrath of Khan*, starship Enterprise's science officer Mr. Spock is in isolation because he has a lethal infection which would wipe out the entire crew should he leave the confinement and seek a cure for himself. Captain Kirk witnesses his decision to stay put and sacrifice himself, the encounter encapsulated in the legendary exchange [49]:

*Mr Spock*: The needs of the many outweigh…

*Captain Kirk*: … the needs of the few.

*Mr Spock*: Or the one.

This is a fine cinematic moment and a priceless utilitarian confession of faith—but it is also blatantly elitist. Elsewhere in the chronicles, Enterprise is often under attack, and the intercom informs us that there are ‘casualties on all decks’ or that ‘contact with the landing party has been permanently lost’. The ones who have perished on the demolished decks or gone missing during the ill‐designed scout‐outs have definitely not had the luxury of Mr. Spock's autonomous and noble choice. They have gone where no one has gone before on their superiors' orders and drawn the short stick. They are dispensable, much against the Kantian imperative of treating humanity in every person as an end in itself [50, p. 38].

In these circumstances, no one even has a right to die, although many have the duty to perish at the altar of the greater good—hence the title of my treatise, echoing the classic *Alien* marketing line ‘In space no one can hear you scream.’ An individual may be ordered into life‐threatening tasks— repairing vessels, defending them—and when their best‐by date has passed, may face a *Brave New World* type fate: a quiet summons to the system's involuntary euthanasia facilities, thereby becoming an object of its biopower [51, pp. 135 ff.]. As for voluntarily giving up one's life in response to intolerable conditions or existential fatigue, that will not be an option when every body is needed for the survival of the exploration party. Out there no one has the right to die.^13^

## 11 Eventual Bliss as an Excuse

Not having the right to die is not, of course, particularly high up on people's list of worries even down here on Earth. It serves, however, as a symbol of the totalitarian nature of life in space. The distances require considerable or complete self‐sufficiency, and strict discipline with no waste or rebellion will probably be regarded as an efficient way to meet the challenge. Individual travellers will not be the masters of their own time, occupations, reproduction, or ultimately even the continuation or cessation of their existence.

Champions of space exploration can admit the validity of these concerns but argue that the harsh conditions and adjacent sacrifices will eventually be justified by the rewards that await successful exploration parties. The quality of life on Mars and further out will, they can claim, be much higher than we are used to down here, because of the endless resources available to the immigrants. Besides, the survival of the human species may in and of itself be a goal worth pursuing [6]. How living conditions would improve or deteriorate on other planets is a matter of pure speculation. What we could ask, though, is this: if huge improvements could be made in unknown and unpredictable environments on faraway exoplanets, what stops us from making them here? The resources are available if only we could distribute them justly. Or reversely: if we cannot prevent ourselves from degrading this plentiful habitat, what would our chances elsewhere be?

These factual prospects and risks cannot be settled by philosophical arguments. Their moral bearing, on the other hand, can be conceptually probed. Can the eventual bliss of unspecified future people legitimate the totalitarian hardship encountered by the interim generations of space explorers? The original colonists can give their consent to the proceedings, but the rest are serving the initial quest involuntarily. Jesuit morality and Leninist consequentialism hold that very good ends justify even the worst means [52, 53], but these are deservedly unpopular views. Most ethical theories demand at least a modicum of prefiguration—adjusting the means so that they are compatible with the ends. The Kantian humanity principle is an example of this but there are others, also within teleological and consequentialist doctrines [54, 55].

In conclusion, I would say that space exploration in the sense of colonizing other worlds is an irrational and immoral endeavour [56]. The sooner the ideas of travelling into the stars are abandoned the better.

## Conflicts of Interest

The author declares no conflicts of interest.

## Endnotes

1 In the article, the views are presented with their context, clockwise from the top left, conservative (tradition), liberal (capability), radical (utility), and progressive (care).

2 That the car is self‐driving is just a mildly alienating detail here. Spaceships can be controlled by qualified crew members, just like our ride could be conducted by a professional driver. On longer outer‐space voyages, though, automation would probably play a focal role, hence the emphasis on self‐driving from the outset.

3 ‘Nor was this appropriation of any parcel of land, by improving it, any prejudice to any other man, since there was still enough and as good left, and more than the yet unprovided could use. So that, in effect, there was never the less left for others because of his enclosure for himself. For he that leaves as much as another can make use of, does as good as take nothing at all. Nobody could think himself injured by the drinking of another man, though he took a good draught, who had a whole river of the same water left him to quench his thirst. And the case of land and water, where there is enough of both, is perfectly the same.’ [10], Book II, Chapter V, Paragraph 22, p. 132.]

4 I am continuing to use the words ‘we’, ‘our’, ‘us’, and so on here without the quotation marks, but they still refer exclusively to my liberal and radical target audience, unless otherwise stated.

5 Not to mention ‘our’ commitment to liberal and utilitarian values and tenets.

6 Interestingly, life, too, can be seen as a illness that should not be spread to outer space [32].

7 The limitation here is purely technical and stipulative. Other species and formations are equally worthy of moral consideration. Addressing them all at once would, however, be cumbersome and draw the attention away from the primary ‘liberal’ and ‘radical’ concern for humankind and its plight.

8 In the sense that I have stated, ‘liberal’ and ‘radical’ assumption.

9 ‘In this and most other civilised countries, for example, an engagement by which a person should sell himself, or allow himself to be sold, as a slave, would be null and void; neither enforced by law nor by opinion. The ground for thus limiting his power of voluntarily disposing of his own lot in life, is apparent, and is very clearly seen in this extreme case. The reason for not interfering, unless for the sake of others, with a person's voluntary acts, is consideration for his liberty. His voluntary choice is evidence that what he so chooses is desirable, or at least endurable, to him, and his good is on the whole best provided by allowing him to take his own means of pursuing it. But by selling himself for a slave, he abdicates this liberty; he foregoes any future use of it beyond that single act. He therefore defeats, in his own case, the very purpose which is the justification of allowing himself to dispose of himself. He is no longer free; but is thenceforth in a position which has no longer the presumption in its favour, that would be afforded by his voluntarily remaining in it. The principle of freedom cannot require that he should be free not to be free. It is not freedom, to be allowed to alienate his freedom.’ [37]

10 My paternal great‐grandparents, originally from Finland, were married in Houghton, Michigan, where they met. Some years later, they packed up their American‐born sons and returned to the homeland, making me, so to speak, a third‐generation Finnish American. Even had they stayed, however, the children and their children's children could have made the return trip at any time. No one was ever stuck in Houghton.

11 Reflections on polar expeditions and Antarctic stations suggest that the harsh environment and isolation may necessitate hierarchical decision‐making to ensure group survival and mission success. It makes sense that such settings prioritize unity and rapid decision‐making over democratic processes. In maritime history, exploration ships—hence my earlier reference to Santa Maria, Pinta, and Niña—traditionally operated under rigid command structures due to the challenges of managing crews in isolated, high‐risk settings. Space is a logical continuum to these environments.

12 I am making free use of the fictional here because that is all that the opposition has to show for its case—rosy visions of democratic family life on board the various Starfleet spaceships of *Star Trek* and similar sagas.

13 To put this differently, in the language of my methodological starting point, the point here is twofold. (i) My ‘we’—the liberals and radicals of my starting point‐‐are uncertain about the human entitlement to die at will. Autonomy is a strong ethical motivator but there are uncertainties that cause hesitation and command caution. Yet both ‘liberals’ and ‘radicals’ agree that the question should remain open—it would be unacceptable to close the door and say that no one, ever, has a right to die. (ii) The conditions during longer space travels, I suggest, might—or even probably would—close that door. Therefore, my liberals and radicals should not accept the development lightly. The denial of a right to die—or its tangible possibility—provides an esoteric‐sounding but solid reason against sustained space travel.
